# Perspectives of Health Care Staff on Predictors of Success in a Food Prescription Program: A Qualitative Study

**DOI:** 10.5888/pcd20.220178

**Published:** 2023-01-12

**Authors:** John Wesley McWhorter, Jennifer N. Aiyer, Nalini Ranjit, Jack Toups, Esther Liew, Jemima C. John, Shreela V. Sharma

**Affiliations:** 1 The University of Texas Health Science Center at Houston, School of Public Health, Health Promotion and Behavioral Sciences, Houston, Texas; 2 The University of Texas Health Science Center at Houston School of Public Health, Department of Epidemiology, Human Genetics and Environmental Sciences, Houston, Texas; 3 The University of Texas Health Science Center at Houston, John P and Katherine G McGovern Medical School, Medical Education, Houston, Texas; 4 Houston Food Bank, Houston, Texas; 5 The University of Texas Health Science Center at Houston, John P and Katherine G McGovern Medical School, Family and Community Medicine, Houston, Texas

## Abstract

Partnerships between food prescription programs and food banks can address food insecurity and support health; however, few studies have examined the experience and perceptions of health care partners about these programs. Our objective was to analyze secondary qualitative data from clinicians and clinic staff involved in implementing a food prescription program in Houston, Texas. We collected data from 17 health care clinics from May 2018 through March 2021 to learn how implementation of the food prescription program was perceived, and we received 252 responses. Principal themes were the importance of a value-based care strategy, patient and food pantry barriers to success, the importance of interorganizational care coordination, and the need to integrate food prescriptions into clinic workflow. Insights of clinicians and clinic staff on implementation of food prescription programs can inform program development and dissemination.

**Summary**

**What is already known on this topic?**

Food prescription programs are increasing rapidly nationwide through health care clinic–community partnerships to reduce household food insecurity and improve health outcomes.

**What is added by this report?**

We surveyed clinicians and clinic staff in several diverse health care clinics about their experience with a food prescription program that involved collaboration between clinics and food banks.

**What are the implications for public health practice?**

Careful planning of collaboration among clinics, food banks, and food prescription programs could promote patient participation to improve health outcomes and reduce food insecurity. Collaboration will require thoughtful care coordination, communication, data sharing, and education.

## Objective

Food insecurity affects approximately 10.5% of households in the US, 13.1% in Texas, and 16.3% in the Greater Houston area (1,2) and is strongly correlated with the prevalence of chronic disease (3). Food prescription programs are gaining popularity as an evidence-based strategy to reduce food insecurity (4). Emerging partnerships between health care clinics and food banks leverage established community points of contact (eg, food banks, food pantries) to provide healthy food to those in need; these partnerships have demonstrated health improvement outcomes (5). Success of such partnership programs is largely dependent on staff referral and care coordination; however, few data exist on how clinic staff perceive such partnerships (6). Since 2018, the Houston Food Bank (HFB) has operated a food prescription program, Food Rx, across 21 health care clinics in the Greater Houston area. Our study examined the perceptions of clinic staff about the importance of the program and challenges they encountered in program implementation. Our objective was to determine what clinicians and other clinic staff members perceived as the benefits of the HFB food prescription program and the barriers encountered in implementing it.

## Methods

We conducted a retrospective secondary analysis of the data HFB collected from May 2018 through March 2021. Electronic surveys were sent by HFB to all clinic staff in 21 participating clinics (federally qualified health centers, charity clinics, health systems, a city health department, and an academic health center) involved in implementing HFB’s food prescription program, HFB Food Rx; 17 responded (81% response rate at the clinic level) (Figure). A convenience sample of 252 health care staff members in the 17 organizations responded to the survey. One open-ended question was asked in the survey: “Briefly (in 250 words or less) provide qualitative feedback related to progress of the Food Rx program and partnership with the Food Rx program, including feedback from participants about Food Rx and ways to improve Food Rx for participants.” Clinic partners gave consent for data use in our research as part of HFB partnership agreements. The primary investigator (J.W.M.) used de-identified data to review the open-ended question and response frequency and to create a preliminary codebook. Response text was then assigned to existing codes with modification as needed to capture emerging themes by using constant comparative methodology (7). Subsequently, 2 additional investigators (J.A., J.T.) blind-coded data by using the codebook as a reliability check. Any code discrepancies were resolved by consensus among the 3 investigators (J.W.M., J.A., J.T.). Data were analyzed in June 2021 by using Dedoose version 9.0.15 (Dedoose: SocioCultural Research Consultants, LLC).

**Figure:**
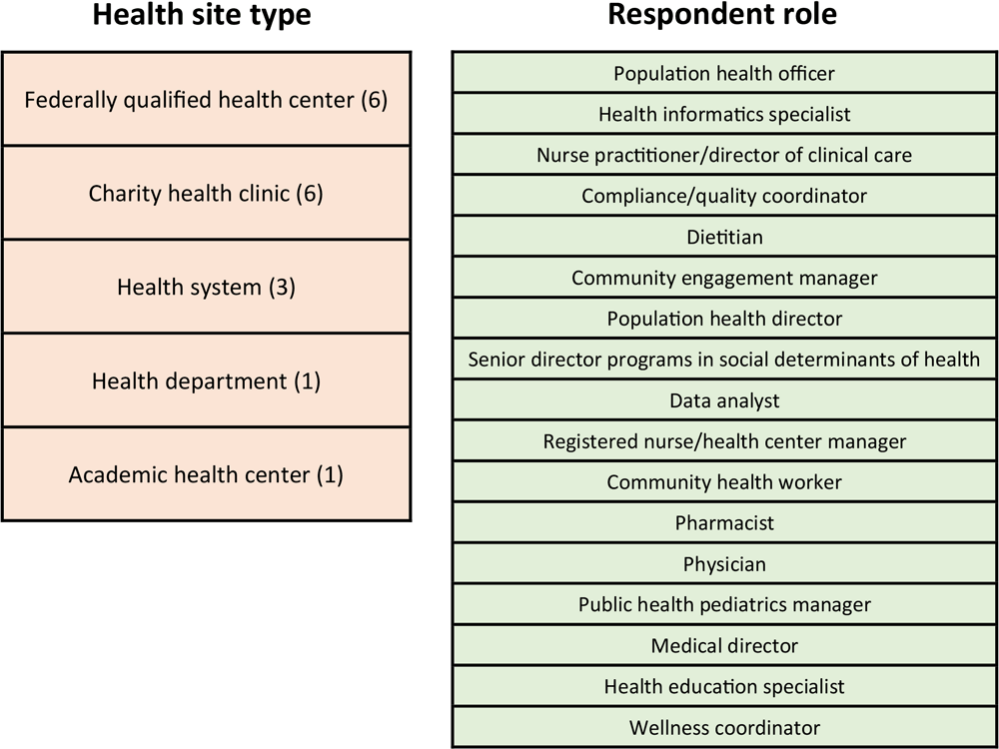
Description of clinics and clinic staff that responded to a survey to assess experience and perceptions of a food prescription program, Food Rx, operated by the Houston Food Bank. The first column lists the 5 types and number of clinics that participated in the survey. The second column lists the 17 job titles of respondents. Houston, Texas, May 2018 to March 2021.

This study was part of a parent study to assess the clinical impact of the HFB food prescription program. The study was approved by The University of Texas Health Committee for Protection of Human Subjects.

## Results

We identified 4 broad areas of experience and perception among survey respondents (Table). The first was the importance of having a value-based care strategy and the role of food prescription programs in that strategy to address food and health needs in populations vulnerable to food insecurity. The second area was patient and food pantry barriers to success in implementation of Food Rx. The third area was the importance of coordinated patient care within and between organizations to implement an effective and standardized partnership-based food prescription program. Coordination included recognition of the value of establishing consistent communication channels between clinic and patients. The fourth area was the need for improved within-clinic workflow integration and quality improvement metrics for clinicians to understand their current performance in screening and responding to patient needs associated with social determinants of health. Respondents also noted the particular importance of food prescription programs during the COVID-19 pandemic.

**Table:** Clinician Comments About Issues Related to Food Prescription Programs, Houston Food Bank, Houston, Texas, May 2018 to March 2021

Theme	Clinician remarks
Importance of having a value-based care strategy	“Now, when the patient states that the reason behind their condition is not being able to afford healthy foods, the providers are able to direct them to the CAP [community assistance program] coordinator and the patients feel like their health is being valued. Since we did not have experienced personnel that were able to assist our patients in signing up for the food stamps and other resources available to the patients, patients are extremely happy that they can walk into the clinic and have their needs met.”
	“Food Rx helps with participants to use their new knowledge about healthy eating by providing them with fruits and vegetables to go home and eat more healthy. It helps relieve the monetary burden and encourage them to try fruits and vegetables that otherwise they would not be able to buy.”
Patient and food pantry barriers to success	“. . . long waiting hours or time, sometimes informed after waiting that markets ran out of food; we are also having some patients with challenges going themselves because of work, so working to try to get them to write another shopper on their behalf when they register.”
	“Participants expressed dissatisfaction with the food options [and] amounts of vegetables available, also noted that if they got there later in the day, the selection was also more limited. One market also had a lot of sweets and sugary foods.”
	“Patients still struggling with transportation, which is limiting the number of visits to the pantries.”
Importance of inter-organization care coordination	“We have noticed that pantry utilization has been low, and we would like to look into ways to encourage participants to use pantries more.”
	“Our partnership [with the markets] and [the food bank] has grown with the past year, we are learning how to better serve our patient population together to make this a more active and functional program for both the clinic and patients.”
Needed workflow integration	“We are in the process of updating workflows and incorporating our Health Educators (at each location) to assist with Program Enrollment, data entry, and patient/client follow up.”
	“. . . working with each clinic to identify patients, I expect this process to get better with time. I do run into issues, if I have a full schedule and time is limited, but I do the best I can in enrolling patients.”
	“From November to December efforts were focused on training the staff that will register patients into Food Rx, which are care coordinators. . . . It is our hope that as more staff see that the referring and enrollment process of Food Rx is less complex than they think, we will have more patients enrolled.”

## Discussion

The perspectives of clinicians and clinic staff in collaborative food prescription programs are critical to understanding program shortcomings and guiding implementation and dissemination design to improve participation and drive overall program success. A consistent theme that emerged from our survey was the perception that a food prescription program is an effective value-based care strategy to address social determinants of health, indicating a positive shift of clinicians’ thinking toward offering such programs as part of the standard of care for patients who are food insecure and struggling with diet-related diseases. A recent systematic review and meta-analysis (5) demonstrated a positive effect of food prescription programs on health outcomes and the desire of clinic staff to continue these programs. As seen in our study, future sustainability efforts need to consider reimbursement for these programs by health insurance companies (8).

Challenges identified by participating clinic staff for program success included the variability of fresh food availability at the food pantry. Although food quality is important to food banks, a recent qualitative study of food bank leadership found that maintaining sufficient stocks of a variety of fresh produce and refrigerated items poses a challenge for food pantries that historically provided only shelf-stable foods (9). This challenge may be exacerbated by other barriers such as patient lack of transportation (10,11). Program success may depend on ensuring a sufficient inventory of fresh produce, maintaining timely restocking practices at pantries, and minimizing patients’ personal barriers.

Additionally, respondents emphasized the importance of interorganizational care coordination, which was reported in prior qualitative research on clinician perspectives on food bank–food prescription partnerships (12). Currently, there is no warm referral (having clinic staff contact another agency or clinician on the client’s behalf) or standardized reminder across clinicians and food pantries to facilitate prescription redemption among patients. In the absence of referrals and reminders, a substantial proportion of patients may fail to redeem their prescriptions, use prescriptions within the 6-month period when the prescriptions are valid, or ever return to the clinic for follow-up.

Finally, we identified clinic workflow integration and use of quality metrics around food prescription programs as important predictors of implementation success. These 2 processes involve developing and training clinic staff in detailed clinic workflow on who will be screened for food insecurity and how, what the clinic response will be to a positive screen (eg, food prescription program referral), and potentially integrating food prescriptions into regular patient care for food-insecure patients. Other studies have reported barriers to program implementation resulting from poor workflow integration, including lack of consistent screening for food insecurity resulting from time constraints and inadequate training of clinic staff (10,13). Furthermore, developing and implementing quality improvement metrics in health care for social determinants of health need to be considered. Measures of the Healthcare Effectiveness Data and Information Set (HEDIS) help clinicians understand their current performance in key areas as well as plan quality improvement efforts. HEDIS measures encompass a number of different health care processes and quality areas, and most recently the National Committee for Quality Assurance (NCQA), the body governing HEDIS measure development, proposed similar quality measures for social determinants of health (14).

Clinicians noted the importance of maintaining communication with patients during the COVID-19 pandemic. In the initial phase of the pandemic, issues related to the food supply chain and unemployment increased rates of food insecurity (15), and lockdowns caused widespread disruption. These conditions reduced access to health clinics and altered food pantry distribution; however, Food Rx pivoted and remained continuously active, which was important to maintaining communication about the program among clinic staff, food pantries, and patients. As one health care organization noted, “HFB has been great at communicating with us and letting us know what their plans [are] and have offered their assistance to us as well. We are very thankful for their partnership, and hope to continue this at our other sites.”

Our study had limitations related to the evaluation of clinician feedback. We used a convenience sample rather than a random sample, which may have resulted in selection bias. The time point of survey administration varied across different clinics, and our evaluation approach was not guided by a validated framework, which could strengthen future studies. However, our scaled evaluation across 21 health care organizations of various types, and a high response rate at the clinic level with a wide range of clinic respondents, provided important feedback related to produce prescription implementation with strong external validity.

In conclusion, our study reports perspectives of clinicians and clinic staff in diverse health care settings on the benefits of food prescription programs for patients, as well as challenges and facilitators to program implementation. Food prescription programs are gaining popularity nationwide, and our study provides insight into successful implementation of such programs. Future studies should incorporate a mixed-methods approach guided by a validated framework to obtain regular input from implementation partners.
